# A novel approach to detect hot-spots in large-scale multivariate data

**DOI:** 10.1186/1471-2105-8-331

**Published:** 2007-09-11

**Authors:** Jianhua Wu, Keith M Kendrick, Jianfeng Feng

**Affiliations:** 1 Department of Computer Science, Warwick University, Coventry CV4 7AL, UK; 2 Cognitive and Behavioural Neuroscience, The Babraham Institute, Cambridge CB2 4AT, UK; 3 Department of Mathematics, Hunan Normal University, 410081 , PRoC

## Abstract

**Background:**

Progressive advances in the measurement of complex multifactorial components of biological processes involving both spatial and temporal domains have made it difficult to identify the variables (genes, proteins, neurons etc.) significantly changed activities in response to a stimulus within large data sets using conventional statistical approaches. The set of all changed variables is termed hot-spots. The detection of such hot spots is considered to be an NP hard problem, but by first establishing its theoretical foundation we have been able to develop an algorithm that provides a solution.

**Results:**

Our results show that a first-order phase transition is observable whose critical point separates the hot-spot set from the remaining variables. Its application is also found to be more successful than existing approaches in identifying statistically significant hot-spots both with simulated data sets and in real large-scale multivariate data sets from gene arrays, electrophysiological recording and functional magnetic resonance imaging experiments.

**Conclusion:**

In summary, this new statistical algorithm should provide a powerful new analytical tool to extract the maximum information from complex biological multivariate data.

## Background

Increasingly, experiments in many areas of biological research simultaneously record activity changes in hundreds or even thousands of variables (i.e. channels, cells, genes, proteins etc) over a time window **T **[1-5]. Due to both internal and external noise, the recorded activity is stochastic. Traditionally all collected data variables are subjected to statistical analysis although, in most cases, not all of them change in response to applied stimuli [6]. Including large numbers of non-responsive variables in the analysis can simply bury the true information carried by a small number of responsive ones leading to the erroneous conclusion that no changes have occurred. In other words, due to the current technology development in biology we often face too much rather than too little data and paradoxically this may sometimes actually impose constraints which prevent us from detecting important information patterns contained within it [7]. In this way important information can be lost. It is therefore of crucial importance to find a way of first filtering out non-responsive variables before performing any further statistical analysis [8].

In [3] we have already proposed a way to handle this problem. MANOVA [9] is applied to analyze data collected from multi-electrode array electrophysiological recordings of activity changes in ensembles of 100 or more different neurons. To assess whether there are significant changes in activity during exposure to a defined sensory stimulus, it is proposed that we have to look at the significance score (a quantitative value corresponding to the significance of the data) of each subset of all of them. Intuitively, it is argued in [3] that the highest value of the significance score corresponds to the area where changes occur. It can readily be seen that this approach could be easily generalized to other types of multivariate biological data such as from gene microarrays or from brain imaging experiments where application of conventional statistical approaches may seriously limit identification of important significant changes. It could also play an important role in the development of systems biology approaches to modeling and understanding complex functional units of biological activity.

However, despite the fact that the approach is interesting and promising, all results presented in [3] are numerical and lacking a rigorous theoretical treatment. On the other hand, the algorithm proposed in [3] is actually an NP hard problem: it requires calculation of the significance score for every subset of the recorded area (variables) and therefore involves considerable computing power for large data sets which makes analysis difficult and time-consuming, if not impossible. In the current paper our primary purpose is to address the following issues:

1. Does the area (set of variables) containing the highest values of significance scores correspond to that where actual changes occur?

2. Does the significance score vanish when more and more non-responsive units are included in the analysis?

3. Can we avoid calculating the significance score of all subsets, i.e. solve the NP hard problem?

4. Can the solution to the problem offer more effective analysis of complex biological multivariate data sets?

We first address these issues in a theoretically treatable model. In general, the set with the highest significance score does not coincide with, but is a subset of, the changed area. However, a single variable exhibits different properties dependent upon whether it belongs to the changed area or not. Based upon this difference in properties exhibited by variables we propose the application of an easily implemented algorithm. This algorithm, which we have called HOTTOR (**hot**-spot detec**tor**), enables us to find the hot-spot and its complexity is only proportional to the total number of variables. Increasing the number of non-responsive variables does not reduce the significance score.

Another interesting finding from our results is the advantage of a system which contains negatively correlated variables compared with one that is positively correlated. It is concluded that the significance score is a decreasing function of the correlation between variables, i.e. it is easier to detect the changes in spatio-temporal patterns if the system is negatively correlated. This also supports one of our long-term working hypotheses that brain neural networks are negatively correlated [10,11] and which we have recently confirmed using multi-array recording approaches in sensory systems [12].

Our approach is first tested on simulated data. It is shown that the HOTTOR algorithm can successfully single out hot-spot variables. We have then applied it to a few examples of biological data sets, including multi-electrode array recording of local field potentials from the brain, gene microarray data and functional magnetic resonance imaging (fMRI) of the brain. In all cases we find that the HOTTOR algorithm outperforms the conventional ANOVA algorithm or, the most commonly used statistical software *Statistical Parametric Mapping *(SPM) for the case of fMRI data.

## Results

**Figure 1:**
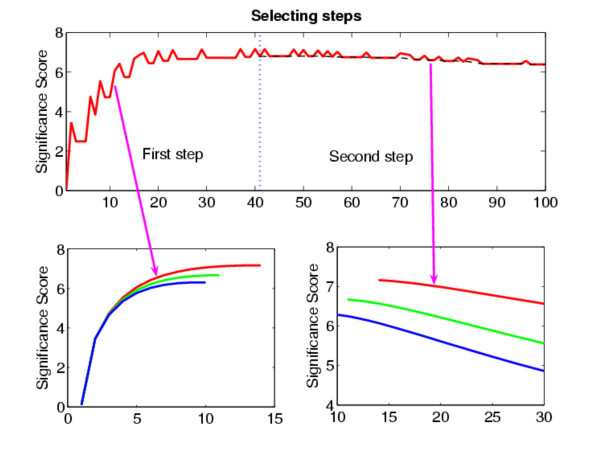
Detecting of the hot-spot for different values of ρ based upon HOTTOR. Upper panel, a trajectory of our selection is presented. Dashed line in the second step is *S*(*A*_*q*+ _∪ {*i*_*r*_}). In the first step, we simply find a subset of the hot-spot *A*_*q*_, relying on the property of the increment of the score. In the second step, a reference is introduced. Bottom panel, from upper to bottom, *ρ *< 0, *ρ *= 0 and *ρ *> 0. Bottom left, the set *A*_*q *_is detected. When *ρ *< 0, *A*_*q *_= {*1*, *2*, ..., *14*}. Bottom, right, the hot-spot is successfully detected for all cases.

Fig. 1 presents a toy example where the HOTTOR algorithm has been applied. The upper panel shows the trajectory of our selection procedure of the HOTTOR algorithm, when *ρ *< 0. The significance score increases when we complete our calculation of 41 variables. We then compare the significance score of each newly picked variable with that of our reference. It is clearly seen that we have successfully found all the changed variables. We have also tested our algorithm with *ρ *> 0 and *ρ *= 0 cases (bottom left and bottom right panel).

When dealing with real-world data, we have to use statistic quantities to check whether adding a variable will increase or decrease the overall score. This is not easily done analytically but we can resort to numerical calculations.

Next we test our algorithm on data from a total of 90 recording variables generated randomly. The first 30 variables of *x*_*1 *_follow a multivariate normal distribution with mean = 0.3. The first 30 variables of *x*_*2 *_are multivariate normal distributed with mean = 1. The rest of the variables of both *x*_*1 *_and *x*_*2 *_follow a multivariate normal distribution with mean = 0. The covariance matrix is given by

Σ=[[1−0.1⋱−0.11]30×30[−0.01−0.01⋱−0.01−0.01]30×60[−0.01−0.01⋱−0.01−0.01]60×30[1−0.1⋱−0.11]60×60]
 MathType@MTEF@5@5@+=feaafiart1ev1aaatCvAUfKttLearuWrP9MDH5MBPbIqV92AaeXatLxBI9gBaebbnrfifHhDYfgasaacH8akY=wiFfYdH8Gipec8Eeeu0xXdbba9frFj0=OqFfea0dXdd9vqai=hGuQ8kuc9pgc9s8qqaq=dirpe0xb9q8qiLsFr0=vr0=vr0dc8meaabaqaciaacaGaaeqabaqabeGadaaakeaacqqHJoWucqGH9aqpdaWadaqaauaabeqaciaaaeaadaWadaqaauaabeqadmaaaeaacqaIXaqmaeaaaeaacqGHsislcqaIWaamcqGGUaGlcqaIXaqmaeaaaeaacqWIXlYtaeaaaeaacqGHsislcqaIWaamcqGGUaGlcqaIXaqmaeaaaeaacqaIXaqmaaaacaGLBbGaayzxaaWaaSbaaSqaaiabiodaZiabicdaWiabgEna0kabiodaZiabicdaWaqabaaakeaadaWadaqaauaabeqadmaaaeaacqGHsislcqaIWaamcqGGUaGlcqaIWaamcqaIXaqmaeaaaeaacqGHsislcqaIWaamcqGGUaGlcqaIWaamcqaIXaqmaeaaaeaacqWIXlYtaeaaaeaacqGHsislcqaIWaamcqGGUaGlcqaIWaamcqaIXaqmaeaaaeaacqGHsislcqaIWaamcqGGUaGlcqaIWaamcqaIXaqmaaaacaGLBbGaayzxaaWaaSbaaSqaaiabiodaZiabicdaWiabgEna0kabiAda2iabicdaWaqabaaakeaadaWadaqaauaabeqadmaaaeaacqGHsislcqaIWaamcqGGUaGlcqaIWaamcqaIXaqmaeaaaeaacqGHsislcqaIWaamcqGGUaGlcqaIWaamcqaIXaqmaeaaaeaacqWIXlYtaeaaaeaacqGHsislcqaIWaamcqGGUaGlcqaIWaamcqaIXaqmaeaaaeaacqGHsislcqaIWaamcqGGUaGlcqaIWaamcqaIXaqmaaaacaGLBbGaayzxaaWaaSbaaSqaaiabiAda2iabicdaWiabgEna0kabiodaZiabicdaWaqabaaakeaadaWadaqaauaabeqadmaaaeaacqaIXaqmaeaaaeaacqGHsislcqaIWaamcqGGUaGlcqaIXaqmaeaaaeaacqWIXlYtaeaaaeaacqGHsislcqaIWaamcqGGUaGlcqaIXaqmaeaaaeaacqaIXaqmaaaacaGLBbGaayzxaaWaaSbaaSqaaiabiAda2iabicdaWiabgEna0kabiAda2iabicdaWaqabaaaaaGccaGLBbGaayzxaaaaaa@90E8@

**Figure 2:**
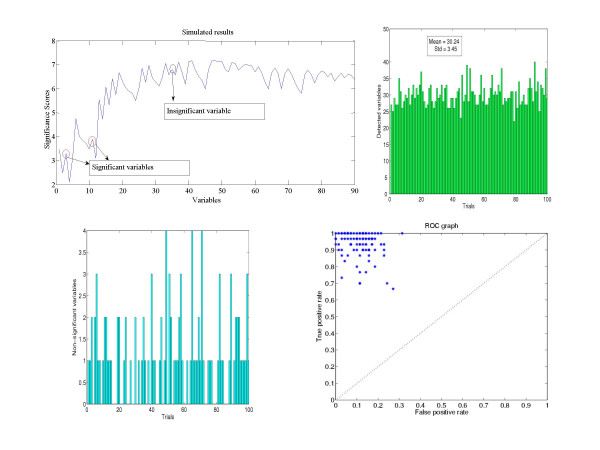
The hot-spot is detected by the HOTTOR algorithm. Upper panel, data are generated according to the covariance matrix Eq. (1). Bottom panel, hot-spots detected by HOTTOR with randomly generated covariance matrices. A total of 100 trials are tested and Bottom panel right depicts the variables wrongly picked by the algorithm.

Different from the toy example above, here we face the situation of having to check statistically whether *S*(*q*)* < S*(*q+1*) or *S*(*q*) > *S*(*q+1*). This is simply carried out in the following way: the confidence intervals for *S*(*q*) and *S*(*q+1*) are constructed at a (*1-α*)% significant level. By comparing the upper limit or lower limit of the confidence intervals of *S*(*q*) and *S*(*q+1*), we can determine whether *S*(*q*) <*S*(*q+1*) or *S*(*q*) > *S*(*q+1*) attain a (*1-α*)% significance level. Now the HOTTOR algorithm is directly applicable to the data set. The following figure shows the detection process. In Fig. 2 upper panel, two variables (indicated by arrows), whose significance scores are smaller than the maximum of all the previous variables, are also statistically different (*P *< 0.05) from them. Hence these two variables are included in *A*_*q*_. In the second step, one variable (indicated by arrow) increases the score, but it is not statistically significant and is not included in *A*_*q*+_. At the end of the process, the hot-spot (total of 30 variables) could be detected by using HOTTOR algorithm.

In all the examples above, the covariance matrix is fixed in advance. We now consider the case that the covariance matrix is generated randomly. In Fig. 2 upper panel right, we depict the detected results for 100 trials. It is readily seen that the detection is an unbiased algorithm, with a variance as indicated in the figure. The bottom left panel shows the variables which are not significant, but wrongly picked up by the HOTTOR algorithm. Fig. 2 bottom right panel is a receiver operating characteristic (ROC) curve [13,14] for true positive rate against false negative rate. It is obvious that the algorithm has very good performance to detect the significantly changed variables. All our results above confirm that HOTTOR is capable of picking up changed variables in a complex spatio-temporal data set.

Finally we have applied our algorithm to biological data collected from electrophysiological microelectrode array recording and gene microarray experiments to assess whether it is more successful in identifying areas of significant change in comparison with conventional approaches (in all cases, *P <*0.05 was considered significant).

**Figure 3:**
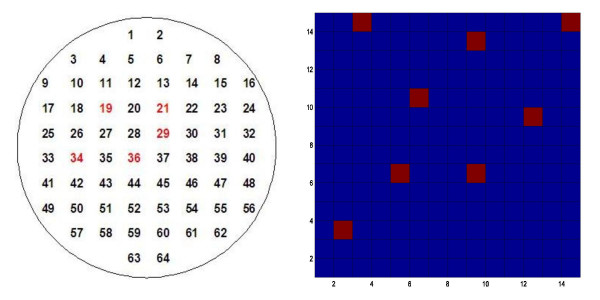
Left, hot-spots detected in 64-channel microelectrode array recordings from sheep temporal cortex during a face recognition task. Right results from a gene microarray experiment identifying significant expression changes in *8 *out of *196 *genes.

In the first example, sheep, under halothane (fluothane) anaesthesia, were implanted with two chronic 64-channel multielectrode arrays in the right and left inferotemporal cortices, respectively. Recordings were made from the unanaesthetised sheep while they performed an operant discrimination task in which different pairs of sheep faces were presented and a correct panel-press response elicited a food reward. The recordings were done by using customized 64 channel multi-electrode arrays of bundled tungsten electrodes each separated by 250 *μ*m. These multi-electrode arrays were implanted chronically under anaesthesia in both hemispheres of the brain in the infero-temporal cortex region. Local event-related LFPs are recorded simultaneously from 128 chronically implanted electrodes. LFP time series data are sampled at 2000 samples/s from around 3 seconds prior to stimulus onset to 3.5 seconds after the stimulus onset in each trial of a session, and stored as 16-bit numbers. Experiments were carried out in accordance with the UK 1986 Animals (Scientific Procedures) Act. A conventional ANOVA test failed to show significant stimulus related changes in any of the 128 electrodes; the maximum computed F-value is 1.12, which is far less than the significance value 1.65. However when HOTTOR analysis was applied significant changes were found in 5 of the 64 channels in the right brain hemisphere (Fig. 3 left panel) and 4 of the 64 channels in the left.

In a second example a set of microarray data [15-17] was obtained from a web source [18]. Microarray data derived from immune B-cells of normal and abnormal human patients were again compared using ANOVA and the HOTTOR algorithm. For simplicity, only 196 genes were analyzed and whereas ANOVA failed to identify significant changes in any of them (the obtained maximum F-value is 0.85 and the significance value is 1.37, the obtained P-value is 0.079) the HOTTOR algorithm identified changes in eight of them (Fig. 3 right panel). The genes showing significant changes can be found in the file [19].

**Figure 4:**
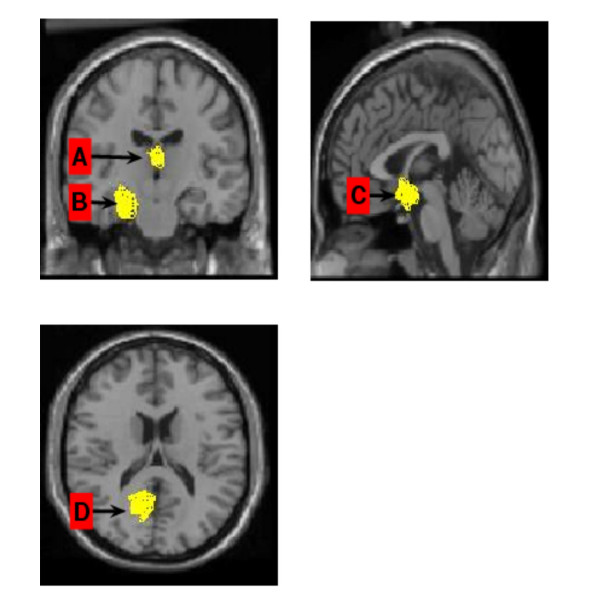
The hot-spots are detected by HOTTOR algorithm in fMRI data (from [13]). Three slices are shown at different orientations and identifying significant altered activity in (A) the medial thalamus, (B) the right amygdala, (C) the head of the caudate nucleus and (D) the right retrosplenial cortex.

In a final example we analyzed data from a functional magnetic resonance imaging (fMRI) experiment [20,21]. The study chosen aimed to identify brain regions showing activation changes while women and men viewed images of people they were romantically in love with [22]. The MRI images were taken every 6 seconds from 20 different slices for 30 s before and during exposure to the test pictures. This gives 72 images before and during the stimulus with each image captured at 79 × 95 pixel resolution. For the purposes of analysis each image pixel was treated as a variable, so we had 7505 variables with 72 replications. To do this we first divided the image into smaller pieces (groups of around 72 pixels). An analysis of the data using the algorithm revealed a number of significant changes associated with seeing the image of the loved-one (Fig. 4). It confirmed significant alterations in activity in the right amygdala, the caudate nucleus and the right retrosplenial cortex as had been reported in the original paper using a standard statistical approach (SPM), although the sizes of the areas were slightly different [22]. However, the HOTTOR algorithm also identified a new area that was changed significantly, the medial thalamus, which is consistent with patterns of changes reported in other regions.

## Discussion and conclusion

We have developed a novel statistical algorithm to detect hot-spots in complex spatio-temporal data sets derived from biological experiments which avoids the problems inherent with current statistical tests where only small numbers of variables are changing within large scale data sets. Such conditions are increasingly encountered in biological experiments that now have the capacity to measure many thousands of variables simultaneously. The algorithm is based upon our theoretical results which found that hot-spots in data are characterized by the non-continuity of the derivative of the significance score. It has been applied successfully to both artificially generated data and real biological data from multi-array electrophysiology, gene array and fMRI experiments. The algorithm is particularly effective in identifying changes in spatio-temporal patterns where variable activity is negatively correlated such as we have found recently using multi-array recordings from brain sensory networks [11,12].

In all of the cases where real biological data has used the algorithm is successful in identifying more significant changes than current standard statistical approaches. Changes of a spatio-temporal pattern in a system of negative correlations between units can be more easily detected than that of positive correlations.

When analyzing the local field potentials data, it requires the data to be stationary; at least the data is stationary in a short time interval. The minimum value for sample size *N *should be larger than the total number of variables. In the present model, the proposed algorithm does not take into account spatio-temporal dependences among the data. The model could include possible geometric or temporal relationships in the data, which would improve the performance and reduce the time cost. The more general case for this method will be investigated in the later publications.

In summary, this new statistical algorithm should provide a powerful new analytical tool to extract the maximum information from complex biological multivariate data.

## Methods

Let *X*_*1 *_= (*x*_*11*_, ..., *x*_*1m*_), *X*_*2 *_= (*x*_*21*_, ..., *x*_*2m*_) be two random vectors with *m *variables in each vector (population). Sampling of the vectors represents their temporal dynamics (electrophysiological recordings) or replications of the experiment (gene microarray data and fMRI images). For population 1, the first *n *variables have a mean of 1, and the rest have a mean of 0. For population 2, the first *n *variables have a mean of *c*, and the rest have a mean of 0. In other words, *n *random variables take different values due to, say, different treatments or stimuli. The hot-spot is {*1*, *2*, ..., *n*}. In the literature [9], the most common way of comparing the difference between two populations is MANOVA. In MANOVA, we first construct a statistical quantity called Wilks' lambda. The hypothesis that *μ*_1_: = EX_1 _(the mean activity of population 1) = *μ*_2_: = EX_2 _(the mean activity of population 2) or not is assessed depending on Wilks lambda. More specifically, let us assume that the covariance matrix is

Σ=[[1ρ⋱ρ1]n×n[ρ1ρ1⋱ρ1ρ1]n×(m−n)[ρ1ρ1⋱ρ1ρ1](m−n)×n[1ρ⋱ρ1](m−n)×(m−n)]
 MathType@MTEF@5@5@+=feaafiart1ev1aaatCvAUfKttLearuWrP9MDH5MBPbIqV92AaeXatLxBI9gBamXvP5wqSXMqHnxAJn0BKvguHDwzZbqegyvzYrwyUfgarqqtubsr4rNCHbGeaGqiA8vkIkVAFgIELiFeLkFeLk=iY=Hhbbf9v8qqaqFr0xc9pk0xbba9q8WqFfeaY=biLkVcLq=JHqVepeea0=as0db9vqpepesP0xe9Fve9Fve9GapdbaqaaeGacaGaaiaabeqaamqadiabaaGcbaGaeu4OdmLaeyypa0ZaamWaaeaafaqabeGacaaabaWaamWaaeaafaqabeWadaaabaGaeGymaedabaaabaacciGae8xWdihabaaabaGaeSy8I8eabaaabaGae8xWdihabaaabaGaeGymaedaaaGaay5waiaaw2faamaaBaaaleaacqWGUbGBcqGHxdaTcqWGUbGBaeqaaaGcbaWaamWaaeaafaqabeWadaaabaGae8xWdi3aaSbaaSqaaiabigdaXaqabaaakeaaaeaacqWFbpGCdaWgaaWcbaGaeGymaedabeaaaOqaaaqaaiablgVipbqaaaqaaiab=f8aYnaaBaaaleaacqaIXaqmaeqaaaGcbaaabaGae8xWdi3aaSbaaSqaaiabigdaXaqabaaaaaGccaGLBbGaayzxaaWaaSbaaSqaaiabd6gaUjabgEna0kabcIcaOiabd2gaTjabgkHiTiabd6gaUjabcMcaPaqabaaakeaadaWadaqaauaabeqadmaaaeaacqWFbpGCdaWgaaWcbaGaeGymaedabeaaaOqaaaqaaiab=f8aYnaaBaaaleaacqaIXaqmaeqaaaGcbaaabaGaeSy8I8eabaaabaGae8xWdi3aaSbaaSqaaiabigdaXaqabaaakeaaaeaacqWFbpGCdaWgaaWcbaGaeGymaedabeaaaaaakiaawUfacaGLDbaadaWgaaWcbaGaeiikaGIaemyBa0MaeyOeI0IaemOBa4MaeiykaKIaey41aqRaemOBa4gabeaaaOqaamaadmaabaqbaeqabmWaaaqaaiabigdaXaqaaaqaaiab=f8aYbqaaaqaaiablgVipbqaaaqaaiab=f8aYbqaaaqaaiabigdaXaaaaiaawUfacaGLDbaadaWgaaWcbaGaeiikaGIaemyBa0MaeyOeI0IaemOBa4MaeiykaKIaey41aqRaeiikaGIaemyBa0MaeyOeI0IaemOBa4MaeiykaKcabeaaaaaakiaawUfacaGLDbaaaaa@978B@

In the matrix, we assume that |*ρ*_*1*_| << |*ρ*|, reflecting the structure in means.

Define

x¯l=∑j=1Nxlj/Nfor l=1,2 andx¯=∑l=12∑j=1Nxlj/(2N),
 MathType@MTEF@5@5@+=feaafiart1ev1aaatCvAUfKttLearuWrP9MDH5MBPbIqV92AaeXatLxBI9gBaebbnrfifHhDYfgasaacH8akY=wiFfYdH8Gipec8Eeeu0xXdbba9frFj0=OqFfea0dXdd9vqai=hGuQ8kuc9pgc9s8qqaq=dirpe0xb9q8qiLsFr0=vr0=vr0dc8meaabaqaciaacaGaaeqabaqabeGadaaakeaafaqabeqadaaabaWaa0aaaeaacqWG4baEaaWaaSbaaSqaaiabdYgaSbqabaGccqGH9aqpdaaeWaqaaiabdIha4naaBaaaleaacqWGSbaBcqWGQbGAaeqaaOGaei4la8IaemOta4ealeaacqWGQbGAcqGH9aqpcqaIXaqmaeaacqWGobGta0GaeyyeIuoaaOqaaiabbAgaMjabb+gaVjabbkhaYjabbccaGiabdYgaSjabg2da9iabigdaXiabcYcaSiabikdaYiabbccaGiabbggaHjabb6gaUjabbsgaKbqaamaanaaabaGaemiEaGhaaiabg2da9maaqadabaWaaabmaeaacqWG4baEdaWgaaWcbaGaemiBaWMaemOAaOgabeaakiabc+caViabcIcaOiabikdaYiabd6eaojabcMcaPaWcbaGaemOAaOMaeyypa0JaeGymaedabaGaemOta4eaniabggHiLdaaleaacqWGSbaBcqGH9aqpcqaIXaqmaeaacqaIYaGma0GaeyyeIuoaaaGccqGGSaalaaa@660E@

where *N *is the number of samplings, and the sampling covariance as:

SSW=∑l=12∑j=1N(xlj−x¯l)(xlj−x¯l)'SSB=∑l=12N(x¯l−x¯)(x¯l−x¯)'
 MathType@MTEF@5@5@+=feaafiart1ev1aaatCvAUfKttLearuWrP9MDH5MBPbIqV92AaeXatLxBI9gBaebbnrfifHhDYfgasaacH8akY=wiFfYdH8Gipec8Eeeu0xXdbba9frFj0=OqFfea0dXdd9vqai=hGuQ8kuc9pgc9s8qqaq=dirpe0xb9q8qiLsFr0=vr0=vr0dc8meaabaqaciaacaGaaeqabaqabeGadaaakqaaeeqaaiabbofatjabbofatjabbEfaxjabg2da9maaqahabaWaaabCaeaacqGGOaakcqWG4baEdaWgaaWcbaGaemiBaWMaemOAaOgabeaakiabgkHiTmaanaaabaGaemiEaGhaamaaBaaaleaacqWGSbaBaeqaaOGaeiykaKIaeiikaGIaemiEaG3aaSbaaSqaaiabdYgaSjabdQgaQbqabaGccqGHsisldaqdaaqaaiabdIha4baadaWgaaWcbaGaemiBaWgabeaakiabcMcaPiabcEcaNaWcbaGaemOAaOMaeyypa0JaeGymaedabaGaemOta4eaniabggHiLdaaleaacqWGSbaBcqGH9aqpcqaIXaqmaeaacqaIYaGma0GaeyyeIuoaaOqaaiabbofatjabbofatjabbkeacjabg2da9maaqahabaGaemOta4KaeiikaGYaa0aaaeaacqWG4baEaaWaaSbaaSqaaiabdYgaSbqabaGccqGHsisldaqdaaqaaiabdIha4baacqGGPaqkcqGGOaakdaqdaaqaaiabdIha4baadaWgaaWcbaGaemiBaWgabeaakiabgkHiTmaanaaabaGaemiEaGhaaiabcMcaPiabcEcaNaWcbaGaemiBaWMaeyypa0JaeGymaedabaGaeGOmaidaniabggHiLdaaaaa@6F4F@

Following on from this, if we always assume that *N *is large enough, so that we do not distinguish between the sampling covariance matrix (mean) and the covariance (mean). Then we introduce Wilks' lambda

Λ=|SSWSSB+SSW|
 MathType@MTEF@5@5@+=feaafiart1ev1aaatCvAUfKttLearuWrP9MDH5MBPbIqV92AaeXatLxBI9gBaebbnrfifHhDYfgasaacH8akY=wiFfYdH8Gipec8Eeeu0xXdbba9frFj0=OqFfea0dXdd9vqai=hGuQ8kuc9pgc9s8qqaq=dirpe0xb9q8qiLsFr0=vr0=vr0dc8meaabaqaciaacaGaaeqabaqabeGadaaakeaacqqHBoatcqGH9aqpdaabdaqaamaalaaabaGaee4uamLaee4uamLaee4vaCfabaGaee4uamLaee4uamLaeeOqaiKaey4kaSIaee4uamLaee4uamLaee4vaCfaaaGaay5bSlaawIa7aaaa@3DBE@

And naturally extend the definition of *Λ *as a function of continuous variables of (*m, n*). As in [3], the significance score of *A *= {*1*, ..., *m*} is given by

S(A)=−[N−m+22]log⁡(Λ(A))/χm2(α)
 MathType@MTEF@5@5@+=feaafiart1ev1aaatCvAUfKttLearuWrP9MDH5MBPbIqV92AaeXatLxBI9gBaebbnrfifHhDYfgasaacH8akY=wiFfYdH8Gipec8Eeeu0xXdbba9frFj0=OqFfea0dXdd9vqai=hGuQ8kuc9pgc9s8qqaq=dirpe0xb9q8qiLsFr0=vr0=vr0dc8meaabaqaciaacaGaaeqabaqabeGadaaakeaacqWGtbWucqGGOaakcqWGbbqqcqGGPaqkcqGH9aqpcqGHsisldaWadaqaaiabd6eaojabgkHiTmaalaaabaGaemyBa0Maey4kaSIaeGOmaidabaGaeGOmaidaaaGaay5waiaaw2faaiGbcYgaSjabc+gaVjabcEgaNjabcIcaOiabfU5amjabcIcaOiabdgeabjabcMcaPiabcMcaPiabc+caVGGaciab=D8aJnaaDaaaleaacqWGTbqBaeaacqaIYaGmaaGccqGGOaakcqWFXoqycqGGPaqkaaa@4D46@

where *χ*^2^_*m *_(*α*) is the upper (100*α*)th percentile of a chi-square distribution with *m *degrees. For any subset A ⊂ {*1*, ..., *m*}, we can define its significant score *S*(*A*) accordingly. Intuitively, as in statistical textbooks, a subset *A *is significantly changed if its score *S*(*A*) is larger than the unity. We arrive at the following conclusions (see additional file 1 for the proof detail of theorem 1).

**Theorem 1 ***For fixed n, Wilks lambda is an increasing function of m > n, it increases to a constant Λ_∞_(c) and is given by*

Λ∞(c)=ρ+(n−1)ρ2−nρ12ρ+(n−1)ρ2+nρ(1−c)24−nρ12
 MathType@MTEF@5@5@+=feaafiart1ev1aaatCvAUfKttLearuWrP9MDH5MBPbIqV92AaeXatLxBI9gBaebbnrfifHhDYfgasaacH8akY=wiFfYdH8Gipec8Eeeu0xXdbba9frFj0=OqFfea0dXdd9vqai=hGuQ8kuc9pgc9s8qqaq=dirpe0xb9q8qiLsFr0=vr0=vr0dc8meaabaqaciaacaGaaeqabaqabeGadaaakeaacqqHBoatdaWgaaWcbaGaeyOhIukabeaakiabcIcaOiabdogaJjabcMcaPiabg2da9maalaaabaacciGae8xWdiNaey4kaSIaeiikaGIaemOBa4MaeyOeI0IaeGymaeJaeiykaKIae8xWdi3aaWbaaSqabeaacqaIYaGmaaGccqGHsislcqWGUbGBcqWFbpGCdaqhaaWcbaGaeGymaedabaGaeGOmaidaaaGcbaGae8xWdiNaey4kaSIaeiikaGIaemOBa4MaeyOeI0IaeGymaeJaeiykaKIae8xWdi3aaWbaaSqabeaacqaIYaGmaaGccqGHRaWkdaWcaaqaaiabd6gaUjab=f8aYjabcIcaOiabigdaXiabgkHiTiabdogaJjabcMcaPmaaCaaaleqabaGaeGOmaidaaaGcbaGaeGinaqdaaiabgkHiTiabd6gaUjab=f8aYnaaDaaaleaacqaIXaqmaeaacqaIYaGmaaaaaaaa@6026@

*The significant score S*(*m*) *decreases as m increases and the hot-spot can be detected using the derivative of Wilks' lambda. In other words, a first-order phase transition occurs and n is the critical point. For fixed value of m, the significance score is an increasing function of n. Furthermore, it is a decreasing function of ρ when all other parameters are fixed*.

**Figure 5:**
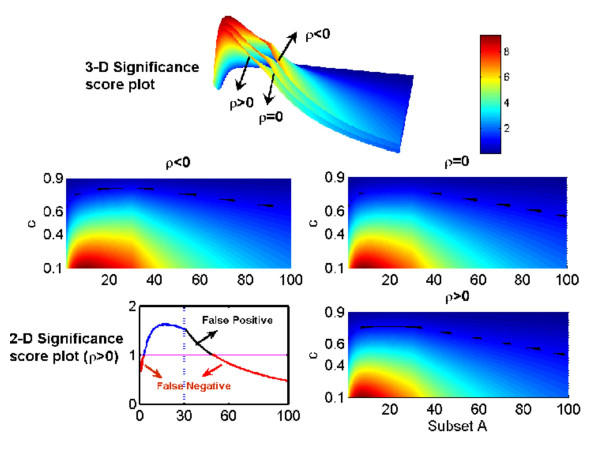
Significance score vs. c and subset *A *= {*1*, *2*, ..., *i*}, *i *= *1*, *2*, ..., *m*. Upper panel is the score vs. *c *and A with different *ρ*. Middle panel and bottom panel, right, is the projection of it onto two-dimensional space. The thick, broken lines indicate the line where the significance score is one. Bottom panel, left, is a section of *c *= 0.7. The horizontal line corresponds to a score of one. The discontinuity of the derivative of the significance score is at *n *= 30, indicated by the vertical, dashed line.

To better elaborate the meaning of Theorem 1, let us consider a few numerical examples with *N *= 200. In Fig. 5, we plot the significance score vs. *c *and *A*, with *n *= 30 (upper panel). The projection of the score onto two dimensions is shown on the middle panel and bottom panel, right. In bottom panel, left, a specific case of *c *= 0.7 with *ρ *> 0 is depicted. As we claim in Theorem 1, the significance score is a decreasing function of *ρ*. With a fixed *c*, it will increase first and then decrease. The hot-spot is not the maximum point of the significance score and changes suddenly when it passes through the point *n *= 30 (Bottom panel, left, indicated by the vertical dotted line). It is easily seen from Fig. 5 that when *m *> *n *= 30, the significance score is a decreasing function. In other words, including data from all the variables in our MANOVA could be misleading in some cases, as we conjectured in [3]. For example, in Fig. 5, *c *= 0.7, if we only include the activity of the first 30 variables in our analysis, we could conclude that there is significant change within the two populations. However, when we include all 100 variables in our analysis we have to say that there is none. Not surprisingly, the significance score is larger when *c *is smaller since the difference between the two populations is greater. From Fig. 5 (upper panel), we see that, unfortunately, the significance score is not always an increasing function of *i *when *i *<*n *= 30. This implies that we cannot detect the hot-spot; here it is {*1*, ..., *n*}, according to the maximum values of the significance score.

From Fig. 5 we observe that the significance score is a decreasing function of *ρ *confirming that it is indeed much easier to detect the changes in a system with negatively correlated variables than with positively correlated or independent ones. Fig. 5 also tells us the difference between using a statistical test of a single variable, which is independent of the covariance matrix, and MANOVA, which is sensitive to the covariance matrix. For example, Fig. 5, bottom left, *c *= 0.7 indicates that if we only test the changes based upon each single variable, then the significance score is always smaller than one. Nevertheless, based upon a group activity (MANOVA), we could assess that there are significant changes between two populations for a subset of all units. This result confirms the benefit of using MANOVA compared with a statistical test based upon a single variable.

Finally detecting the discontinuity of the derivative of the significance score based upon statistical data is not an easy task since the stochastic fluctuations always result in an irregularity in the score. In other words, it is hardly possible to detect the discontinuity of the derivative. We will next consider how to develop a statistical approach to detect the hot-spot.

All results above are established for general case (refer to our homepage for details [23]).

## Algorithm

As we mentioned, the method is developed for the specified biological data, but it is not readily applicable to such data. Because our purpose is not to test the whole set of variables but to detect the variables with significance change. So the first problem we encounter is the representation problem.

Assume we have m variables {*x*_*1*_, *x*_*2*_, ..., *x*_*m*_} with sample size *N*. One option we have is to enumerate all possible combinations of these m variables. For *m *is large, this is known as a NP hard problem. We could have

Total number of combinations = *2*^*m*^

Suppose *m *> 20, then total number of combinations will exceed one million, which is impossible for us to calculate and compare all significance scores. In order to detect the Hot-spot from the whole data set, we have to try other options.

Results from the previous section tell us that

1. The significance score is an increasing function of a subset of the hot-spot. In other words, we can detect part of the all changed set according to whether adding a variable increases or decreases the score.

2. The decreasing rate is different for a changed one compared with an unchanged one. The latter will cause the significance score to decrease faster than the former.

Based upon the observations above we are able to develop the following HOTTOR algorithm. Algorithm and relevant programs can be downloaded freely from [23].

### First Step: Monotonic step

Selecting a variable, say *i*_*1*_, from the whole set {*1*, *2*, ..., *m*}, we then randomly select another one *i*_*2 *_and calculate *S*({*i*_*1*_,*i*_*2*_}). If *S*({*i*_*1*_}) <*S*({*i*_*1*_,*i*_*2*_}), then *i*_*2 *_is one of the changed variables. We continue this procedure and keep the variable if the significance score increases and discard it if it decreases. The monotonic step stops when the significance score reaches its maximum. The obtained set is denoted by *A*_*q*_.

### Second Step: Reference step

After finding the variables which contribute positively to the significance score, we now have to deal with those which do not, but do change their mean values. At this step, we assume that at least one variable which does not change its activity is picked up, say *i*_*r*_. We use the score of this variable as a reference to assess whether a remaining one, say *i*, changes its activity or not. The obtained set is denoted by *A*_*q*+ _⊃ *A*_*q*_. If it changes, i.e., *S*(*A*_*q*+ _∪ {*i*_*r*_}) <*S*(*A*_*q*+ _∪ {*i*}), we then add it to the changed set *A*_*q*+ _= *A*_*q*+ _∪ {*i*}, and continue with our selection. If it does not change, which means *S*(*A*_*q*+ _∪ {*i*_*r*_}) ≥ *S*(*A*_*q*+ _∪ {*i*}), we then simply choose a new variable to check. The above procedure is continued until all the variables are checked. We then have *A*_*q*+ _= {*1*, *2*, ..., *n*}.

## Authors' contributions

JHW – has made substantial contributions to conception and design of the study, analysis and interpretation of data and has been involved in drafting the manuscript. KK – has contributed towards the biological applications of the algorithms, discussed the results and critically revised the manuscript for important intellectual content and has given the final approval of the version to the published. JFF – has made substantial contributions to conception and design of the study, analysis and interpretation of data and has been involved in drafting the manuscript. All authors read and approved the final manuscript.

## Supplementary Material

Additional file 1 Proof of theorem 1. The detailed mathematical proof of theorem 1. Click here for file
