# Supplementary material for: A novel approach to detect hot-spots in large-scale multivariate data
Source: BMC Bioinformatics. 2007 Sep 11;8:331. doi: 10.1186/1471-2105-8-331 (PMC2045117; doi:10.1186/1471-2105-8-331)
Supplement: Additional file 1 — Proof of theorem 1. The detailed mathematical proof of theorem 1. [file 1471-2105-8-331-S1.doc]

**Proof of Theorem 1**

$$\Lambda=\left|\frac{\text{SSW}}{\text{SSB}+\text{SSW}}\right|=\left|\frac{\Sigma}{\frac{(1-c)^{2}}{4}\left[\begin{matrix} 1_{n\times n} & 0_{n\times(m-n)} \\ 0_{(m-n)\times n} & 0_{(m-n)\times(m-n)} \end{matrix}\right]+\Sigma}\right|$$

Therefore, the meaning of$\Lambda$is clear. It compares the difference between the covariance matrix **SSW** with the difference matrix **SSB**. When this difference is big, we say that there are significant changes in the mean of the two populations.

Let us have some more notations:

$$\Sigma_{1}=\frac{(1-c)^{2}}{4}\left[\begin{matrix} 1_{n\times n} & 0_{n\times(m-n)} \\ 0_{(m-n)\times n} & 0_{(m-n)\times(m-n)} \end{matrix}\right]+\Sigma=\left[\begin{matrix} \Sigma_{11} & \Sigma_{12} \\ \Sigma_{21} & \Sigma_{22} \end{matrix}\right]$$

Where

$$\Sigma_{11}=\left[\begin{matrix} 1+\frac{(1-c)^{2}}{4} & & \rho+\frac{(1-c)^{2}}{4} \\ & \ddots& \\ \rho+\frac{(1-c)^{2}}{4} & & 1+\frac{(1-c)^{2}}{4} \end{matrix}\right]_{n\times n};$$

$\Sigma_{12}=\left[\begin{matrix} \rho_{1} & & \rho_{1} \\ & \ddots& \\ \rho_{1} & & \rho_{1} \end{matrix}\right]_{n\times(m-n)};$ $\Sigma_{21}=\Sigma_{12}'$ ; $\Sigma_{22}=\left[\begin{matrix} 1 & & \rho\\ & \ddots& \\ \rho& & 1 \end{matrix}\right]_{(m-n)\times(m-n)}$

Remembering that$\Sigma_{1}=\left|\Sigma_{11}\right|\left|\Sigma_{22}-\Sigma_{21}\Sigma_{11}^{-1}\Sigma_{12}\right|$, and after some detailed calculations, we arrive at:

$$\left|\Sigma\right|=[1+(m-2)\rho+(n-1)(m-n-1)\rho^{2}-n(m-n)\rho_{1}^{2}](1-\rho)^{m-2}$$

And

$$\left|\Sigma_{1}\right|=\left\{\begin{matrix} \begin{matrix} \left[\frac{n((m-n-1)\rho+1)(1-c)^{2}}{4}-n(m-n)\rho_{1}^{2}\right](1-\rho)^{m-2} \\ +[1+(m-2)\rho+(n-1)(m-n-1)\rho^{2}](1-\rho)^{m-2},\begin{matrix} when & m>2n \end{matrix} \end{matrix} \\ \begin{matrix} \left[\frac{n((n-1)\rho+1)(1-c)^{2}}{4}-n(m-n)\rho_{1}^{2}\right](1-\rho)^{m-2} \\ +[1+(m-2)\rho+(n-1)(m-n-1)\rho^{2}](1-\rho)^{m-2}\begin{matrix} when & m\leq2n \end{matrix} \end{matrix} \end{matrix}\right.$$

It is then concluded that Wilks lambda is given by

$\frac{1+(m-2)\rho+(n-1)(m-n-1)\rho^{2}-n(m-n)\rho_{1}^{2}}{1+(m-2)\rho+(n-1)(m-n-1)\rho^{2}+\frac{n((m-n-1)\rho+1)(1-c)^{2}}{4}-n(m-n)\rho_{1}^{2}}$ (3)

When$m>2n$, and

$\frac{1+(m-2)\rho+(n-1)(m-n-1)\rho^{2}-n(m-n)\rho_{1}^{2}}{1+(m-2)\rho+(n-1)(m-n-1)\rho^{2}+\frac{n((n-1)\rho+1)(1-c)^{2}}{4}-n(m-n)\rho_{1}^{2}}$ (4)

When$m\leq2n$.

Eq. (3) and (4) yield some interesting conclusions. First of all, let us check whether the significance score is a decreasing function of *m* when$m>n$, as we have conjectured in [3]. To this end, we first need to calculate the derivatives of Wilks lambda with respect to m, when$m>n$. Denote$B=1+(m-2)\rho+(n-1)(m-n-1)\rho^{2}-n(m-n)\rho_{1}^{2}$, then for $m>2n$

$$\Lambda=\frac{B}{B+\frac{n((m-n-1)\rho+1)(1-c)^{2}}{4}}$$

And

$$\Lambda'=\frac{n^{2}\rho_{1}^{2}(1-c)^{2}(1-\rho)}{4\left[B+\frac{n((m-n-1)\rho+1)(1-c)^{2}}{4}\right]^{2}}$$

For $m\leq2n$

$$\Lambda=\frac{B}{B+\frac{n((n-1)\rho+1)(1-c)^{2}}{4}}$$

And

$$\Lambda'=\frac{n[\rho+(n-1)\rho^{2}-n\rho_{1}^{2}][(n-1)\rho+1](1-c)^{2}}{4\left[B+\frac{n((n-1)\rho+1)(1-c)^{2}}{4}\right]^{2}}$$

Obviously,$\rho<1,\left|\rho_{1}\right|<\left|\rho\right|$, for both cases we conclude that $\Lambda'>0$, which indicates that$\Lambda$is a monotonically increasing function with respect to m, when $m>2n$. Then the significance score$S(A)$ is monotonically decreasing with respect to m.

Secondly we want to check whether the significance score is an increasing function of n when m is fixed. Again, we calculate the derivative of Wilks lambda with respect to n, then for $n<\frac{m}{2}$

$$\begin{matrix} \Lambda'=4\frac{(1-c)^{2}(1-\rho)(n\rho_{1}-1+\rho+n\rho-m\rho)(n\rho_{1}+1-\rho-n\rho+m\rho)}{\left[B+\frac{n((m-n-1)\rho+1)(1-c)^{2}}{4}\right]^{2}} \\ \begin{matrix} & \leq4 \end{matrix}\frac{(1-c)^{2}(1-\rho)(\rho-1++\rho(2n-m))(n\rho_{1}+1-\rho+\rho(m-n))}{\left[B+\frac{n((m-n-1)\rho+1)(1-c)^{2}}{4}\right]^{2}} \\ \begin{matrix} & <0 \end{matrix} \end{matrix}$$

For $\frac{m}{2}\leq n\leq m$

$$\Lambda'=4\frac{(1-c)^{2}(1+(m-1)\rho)(\rho_{1}-1+\rho+(n-1)(\rho_{1}-\rho))(n\rho_{1}+1+(n-1)\rho)}{\left[B+\frac{n((m-n-1)\rho+1)(1-c)^{2}}{4}\right]^{2}}<0$$

For both cases,$\Lambda'<0$, we see that Wilks lambda is a decreasing function with respect to n when m is fixed. Then the significant score $S(A)$ is a monotonically increasing function with respect to n.

Thirdly, for other fixed parameters, we can prove that the significance score is also a decreasing function with respect to$\rho$.

Fourthly, we want to find out how to detect the hot-spot in our setup. Here the hot-spot is$\{1,2,\cdots,n\}$. Let $A=\{1,2,\cdots,i\}$ and$\rho\neq0$. When $i\leq n$ we see

$\Lambda(A)=\frac{1+(i-2)\rho+(n-1)(-1)\rho^{2}}{1+(i-2)\rho+(i-1)(-1)\rho^{2}+\frac{i((-1)\rho+1)(1-c)^{2}}{4}}$ (5)

When$i>n$, we then have an extra term $n(i-n)\rho_{1}^{2}$ in the expression of Λ, i.e.

$\Lambda(A)=\frac{1+(i-2)\rho+(n-1)(i-n-1)\rho^{2}-n(i-n)\rho_{1}^{2}}{1+(i-2)\rho+(n-1)(i-n-1)\rho^{2}+\frac{n((i-n-1)\rho+1)(1-c)^{2}}{4}-n(i-n)\rho_{1}^{2}}$ (6)

We thus conclude that the derivative of $\Lambda(A)$ is not continuous when $i=n$ and$\rho\neq0$. When$\rho=0$, Eq. (5) turns out to be

$$\Lambda(A)=\frac{1}{1+\frac{i(1-c)^{2}}{4}}$$

When $i\leq n$ and

$$\Lambda(A)=\frac{1}{1+\frac{n(1-c)^{2}}{4}}$$

When$i>n$. Again the derivative of $\Lambda(A)$ is not continuous when$i=n$. Summarizing the results above, we have proved Theorem 1.
